# Fiber-optic Fabry–Pérot interferometric accelerometer with composite cavity and temperature calibration for high-temperature and high-pressure applications

**DOI:** 10.1038/s41378-026-01250-z

**Published:** 2026-04-28

**Authors:** Feng Qin, Jiahang Tan, Jiangtao Guo, Zhiqiang Shao, Ning Wang, Jie Zhang, Yong Zhu

**Affiliations:** 1 College of Optoelectronic Engineering, Chongqing University, Chongqing, 400044 China; 2 The 49th Research Institute of China Electronics Technology Group Corporation, Harbin, 150000 China

**Keywords:** Electrical and electronic engineering, Optical sensors

## Abstract

To address the demand for flow-induced vibration monitoring of steam generator heat transfer tubes in pressurized water reactors under high-temperature (350 °C) and high-pressure (17.5 MPa) conditions, a fiber-optic Fabry–Pérot interferometric accelerometer based on a composite Fabry–Pérot cavity structure is proposed. The sensor employs a symmetrically arranged multidirectional cantilever beam and a central proof mass to effectively reduce cross-axis sensitivity. Using a MEMS-based fabrication process, a three-layer sensing chip with a composite cavity is formed, mitigating the temperature drift problem of conventional single-cavity structures under elevated temperatures. A temperature calibration model is further incorporated to improve measurement accuracy. The optical path is folded by a 45° metallic mirror and hermetically sealed by laser welding, ensuring stable operation under high temperature, high pressure, and external mechanical shocks. Experimental results show that the sensor achieves a sensitivity of 4.53 nm/g, a resonant frequency of 7450 Hz, a cross-axis sensitivity as low as 0.281%, and a resolution of 4.4 mg, with an acceleration measurement range of ±238 g at room temperature. Under 350 °C and 17.5 MPa, the sensor exhibited cavity length drift below 0.1 nm during a 60-h stability test, demonstrating reliable dynamic performance and long-term stability in extreme conditions, which provides an effective tool for the continuous safety monitoring of critical heat transfer structures in pressurized water reactors.

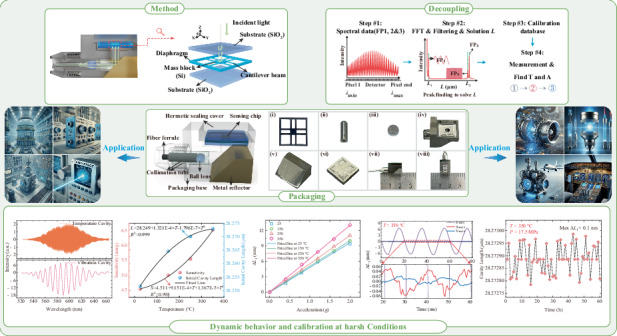

## Introduction

Nuclear energy, as an efficient, clean, and safe form of energy, plays an increasingly important role in the global energy transition^1^. With the growing scale and service life of nuclear power units, higher requirements are being placed on reactor operational safety, among which the structural integrity of steam generator (SG) heat transfer tubes has attracted significant attention. Flow-induced vibration (FIV) caused by coolant flow may lead to local degradation such as wear and gap variation between the tubes and support structures, and may further result in fatigue cracks or rupture of the tube walls, thereby threatening the isolation safety of the primary and secondary circuits^2,3^. Thus, establishing accurate and reliable methods for FIV monitoring is essential for ensuring the long-term stability of heat transfer tubes. The SGs in pressurized water reactors (PWRs) operate in high-temperature (350 °C) and high-pressure (17.5 MPa) coolant environments, which impose stringent requirements on the anti-interference capability and environmental adaptability of sensors^4,5^.

Conventional vibration monitoring technologies largely rely on piezoelectric, capacitive, or MEMS-based accelerometers, but their performance is often constrained under strong electromagnetic interference, high radiation, and elevated temperatures^6,7^. In contrast, fiber-optic Fabry–Pérot (F–P) sensors offer distinct advantages, including immunity to electromagnetic interference, high-temperature resistance, compact structure, and high sensitivity^8–10^. They have been widely employed in measuring various physical quantities, such as pressure^11,12^, strain^13,14^, ultrasound^15^, angle^16^, acceleration^17–21^, interelectrode gap^22^, and physiological signals (e.g., heart rate)^23^, showing broad application prospects in industrial inspection, structural health monitoring, biomedicine, aerospace, and extreme-environment sensing^24^.

Fiber-optic Fabry–Pérot interferometric accelerometers (FPIAs) are highly sensitive to small displacements based on optical cavity interference. Their compactness and ease of integration allow direct installation on key positions of PWR heat transfer tubes, enabling real-time and precise monitoring of FIV. In recent years, the potential of FPIAs has received considerable attention. In 2021, Mahissi et al. ^25^ developed an F–P accelerometer consisting of a single-mode fiber and a SiC diaphragm, achieving a sensitivity of ~370 mV/g, a resonant frequency of 3750 Hz, a cross-axis sensitivity of 11%, and stable operation up to 800 °C. In 2022, Qian et al. ^26^ reported a silicon-based F–P vibration sensor with a resonant frequency of 10.008 kHz and a sensitivity of 2.48 nm/g under 200 Hz excitation, operable in the 20–400 °C range. In the same year, Cui et al. ^27^ fabricated an accelerometer using a diaphragm with simply supported beams via femtosecond laser processing, with a resonant frequency of 2700 Hz, a sensitivity of 20.91 nm/g, and an operating temperature range of 25–1500 °C. In 2024, Wang et al. ^28^ proposed an F–P accelerometer with an aluminum alloy proof mass, achieving a resonant frequency of 2100 Hz, a sensitivity of 4.91 nm/g at 1300 Hz, a resolution of 0.204 mg, and a cross-axis sensitivity of 4.57%. In the same year, Cao et al. ^29^ used femtosecond laser processing of sapphire to fabricate an F–P accelerometer with stable operation from 25–600 °C, a sensitivity of 38.66 nm/g, a resonant frequency of 2446 Hz, and a cross-axis sensitivity of 4.09%. More recently, in 2025, Zhuang et al. ^30^ designed a MEMS-based F–P accelerometer with a sensitivity of 12.397 nm/g at 200 Hz excitation, a resonant frequency of 6624 Hz, a resolution of 14.358 mg, and a cross-axis sensitivity below 6.91%. In the same year, Zhang et al. ^31^ proposed a MEMS chip-based high-sensitivity, wide-band F–P accelerometer. By designing a dual-stage spring resonance structure and introducing in-plane vibration modes, they expanded the sensor’s frequency range. Experimental results show a flat response from 1 to 1780 Hz, with a sensitivity of 11.55 dB re rad/g and cross-axis sensitivity under 1.33%.

Although progress has been made in improving the high-temperature performance of FPIAs, limitations remain in temperature compensation and cross-axis sensitivity control. Most existing sensors employ a single-cavity design using the fiber end face as one reflective surface. This design is susceptible to the bonding process at high temperatures, causing irreversible axial expansion or contraction of the fiber during thermal variations, which reduces repeatability. Furthermore, temperature cross-sensitivity is common, where temperature variations interfere with vibration measurements, degrading accuracy. Existing packaging technologies also lack sufficient high-temperature sealing capability, making it difficult to withstand long-term thermal cycling under high-temperature and high-pressure conditions. More importantly, current designs often suffer from insufficient cross-axis suppression, which limits their robustness in multidirectional vibration monitoring. Under harsh PWR operating conditions, cross-axis sensitivity may be further amplified, thereby compromising measurement accuracy and reliability.

To overcome these issues, this work proposes a high-temperature and high-pressure temperature-calibrated FPIA based on a composite cavity, designed for microvibration monitoring of SG heat transfer tubes in PWR coolant environments. By introducing a glass substrate to form a composite cavity comprising a substrate cavity and an air cavity, the fiber end face is no longer used as a reflective surface. This spatial separation between the fiber and the F–P cavity effectively suppresses cavity length drift caused by thermal expansion, enabling temperature compensation. In the composite cavity, one substrate cavity functions as a temperature cavity for in situ temperature measurement, while the air cavity is dedicated to vibration sensing. A temperature calibration model is incorporated to enhance measurement accuracy. The FPIA has a compact size (20 × 10 × 10 mm), and its sensing diaphragm features symmetrically arranged multidirectional cantilever beams with a central proof mass, fabricated using MEMS wet etching to reduce cross-axis sensitivity. The sensing chip is assembled via direct bonding, and laser welding is applied to ensure high-temperature sealing. This design demonstrates strong environmental adaptability and engineering feasibility for FIV monitoring in SG heat transfer tubes, providing a promising solution for the long-term safety monitoring of critical heat transfer structures in nuclear power plants.

## Sensor structure, operating principle, and mechanical characteristics

### Sensor structure

**Fig. 1:**
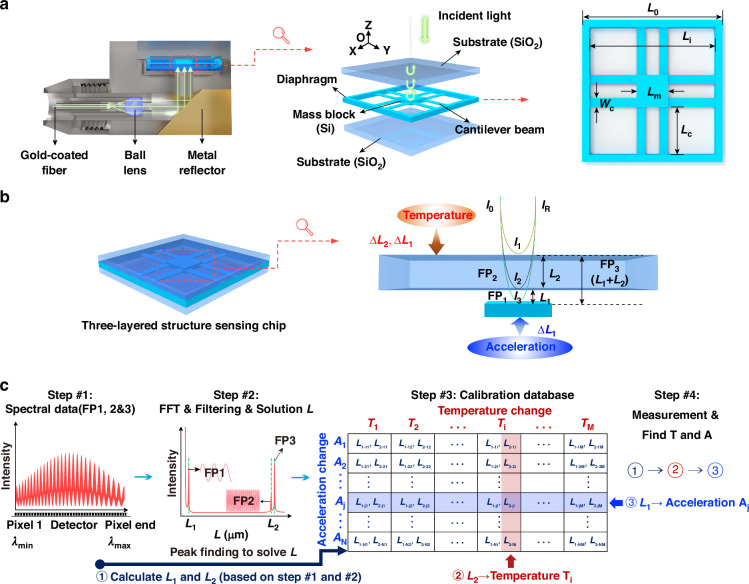
Schematic structure and measurement principle of the FPIA. **a** Schematic of sensor structure. **b** Interference model and data processing workflow. **c** Demodulation of measurement results and calculation of acceleration and temperature

Figure 1a shows the schematic structure of the FPIA, which consists of a packaging base, gold-coated fiber, fiber ferrule, collimation tube, ball lens, metal reflector, sensing chip, and hermetic sealing cover. The sensing chip is composed of two glass substrates and a silicon diaphragm. The diaphragm integrates four symmetrically arranged groups of eight cantilever beams, a central proof mass, and a surrounding support frame. A hollowed-out design in the middle section of each pair of cantilever beams effectively reduces structural stiffness, thereby significantly enhancing the sensor’s response to small acceleration variations.

During operation, the incident light is directed perpendicularly onto the chip structure. Part of the light is reflected between the upper and lower surfaces of the top glass substrate, forming a two-beam interference pattern that constitutes the temperature F–P cavity (FP_2_) with a thickness of *L*₂ = 400 μm. Another portion of the light is reflected between the upper surface of the proof mass and the lower surface of the top glass substrate, forming the vibration F–P cavity (FP_1_) with a thickness of *L*₁ = 25 μm. These two F–P cavities together form a temperature–vibration coupled cavity, denoted as FP_3_ (FP_1_ + FP_2_).

### Temperature–vibration measurement principle of the sensor

As shown in Fig. 1b, when an incident beam with an intensity of *I*₀ passes sequentially through the composite cavity structure, multiple reflections and interferences occur at each interface. First, part of the light is reflected at the top surface of the glass substrate, forming the reflected beam *I*₁. The remaining light enters the temperature cavity and is reflected at the bottom surface of the glass substrate, generating *I*₂. Subsequently, the residual light further enters the vibration cavity and is reflected at the top surface of the proof mass, producing *I*₃. The three beams undergo multi-beam interference, producing the total reflected intensity *I*_R_, which can be expressed as:

1 $${I}_{R}(\lambda )={I}_{1}+{I}_{2}+{I}_{3}-2\sqrt{{I}_{2}{I}_{3}}\,\cos ({\varphi }_{air})-2\sqrt{{I}_{1}{I}_{2}}\,\cos ({\varphi }_{glass})+2\sqrt{{I}_{1}{I}_{3}}\,\cos ({\varphi }_{air-glass})$$

Here, *I*_1_, *I*_2_, and *I*_3_ denote the effective intensities of the three reflected beams. During interference process, the phase shifts of the vibration cavity and the temperature cavity, denoted as *φ*_air_ and *φ*_glass_, are determined jointly by the geometric lengths and refractive indices of the two cavities, and can be expressed as:

2 $${\varphi }_{air}=\frac{4\pi {n}_{air}{L}_{1}}{\lambda },{\varphi }_{glass}=\frac{4\pi {n}_{glass}{L}_{2}}{\lambda },{\varphi }_{air-glass}={\varphi }_{air}+{\varphi }_{glass}$$

Here, *n*_air_ and *n*_glass_ represent the refractive indices of air and glass, respectively; *L*₁ and *L*₂ are the cavity lengths of the vibration cavity and the temperature cavity; and *λ* is the wavelength of the incident light. When the sensing chip is subjected to external acceleration along the Z-axis, the proof mass undergoes a small displacement Δ*L*₁ due to inertia, leading to a change in the vibration cavity and a corresponding shift in the output interference spectrum. By real-time demodulation of this spectrum, external vibration signals can be measured with high accuracy. The temperature cavity, formed by the glass substrate, is insensitive to vibration. Its thickness variation Δ*L*₂ with temperature enables in situ temperature measurement.

As shown in Fig. 1c, the reflection spectrum of the FPIA is collected by a spectrometer, and it represents the superposition of the reflection amplitudes from each reflective surface. In the spectral model, the alternating-current (AC) terms are standard cosine functions with respect to the wavenumber *k* = *2π/λ*. The frequency and phase information of these cosine terms contain the cavity length *L* to be measured. By transforming the intensity model into the wavenumber domain and simplifying, the following expression is obtained:

3 $${I}_{{\rm{R}}}(k)={I}_{{\rm{dc}}}-{I}_{{\rm{ac}}\_1}\,\cos (2{n}_{air}{L}_{1}k)-{I}_{{\rm{ac}}\_2}\,\cos (2{n}_{glass}{L}_{2}k)+{I}_{{\rm{ac}}\_3}\,\cos (2{n}_{air}{L}_{1}k+2{n}_{glass}{L}_{2}k)$$

In this expression, *I*_dc_ denotes the direct-current (DC) term from Eq. (1), while *I*_ac_1_, *I*_ac_2_, and *I*_ac_3_ represent the amplitudes of the AC terms in Eq. (1), corresponding to the single-cavity reflection spectra of FP₁, FP₂, and FP₃, respectively. For simplification, the cosine term of the AC component corresponding to FP₁ is considered, and its Fourier transform can be expressed as:

4 $$\begin{array}{c}{F}_{ac\_1}(\xi )={\int }_{-\infty }^{+\infty }\cos (2{n}_{1}{L}_{1}k){e}^{-i2\pi \xi k}dk\\ =\frac{1}{2}{\int }_{-\infty }^{+\infty }({e}^{i(2{n}_{1}{L}_{1}k)}+{e}^{-i(2{n}_{1}{L}_{1}k)}){e}^{-i2\pi \xi k}dk\\ =\pi \delta (2{n}_{1}{L}_{1}-2\pi \xi )+\pi \delta (2{n}_{1}{L}_{1}+2\pi \xi )\end{array}$$

In this expression, *F*_ac_1_ denotes the Fourier transform result of the cosine term in the reflection intensity of FP₁, and *ξ* is the Fourier transform variable, which is related to the cavity length *L*. *F*_ac_ reaches its maximum at *ξ* = *n*₁*L*₁*/π*, from which the cavity length can be determined as *L*₁ *=* *ξπ/n*₁. The result for FP₂ is obtained in a similar way. The relationships between *L*₁, *L*₂ and the acceleration *A* and temperature *T* can be established through experimental calibration. Once *L*₁ and *L*₂ are obtained, the calibrated results can be used to determine the target values of *A* and *T*.

In high-temperature and high-pressure environments, FPIA is prone to temperature drift due to the fiber’s fixation relying on adhesive materials, which can cause fiber slip and affect the sensor’s stability. To solve this issue, we introduce a glass substrate to isolate the fiber end face from the F–P cavity, effectively eliminating temperature drift and achieving temperature compensation. At the same time, a decoupling algorithm combined with FFT and band-pass filtering is used to separate temperature and vibration signals, accurately calculate cavity lengths, and GPU acceleration and Kalman filtering are employed to improve computation speed and accuracy. Temperature compensation is achieved by establishing a calibration database covering a temperature range of 0–350 °C and an acceleration range of 0–250 g for real-time compensation.

As shown in Fig. 1c, the entire process consists of four main steps: First, spectral data is collected from the FPIA, and the signal is separated using FFT and filtering to calculate cavity lengths *L*_1_ and *L*_2_. Next, a calibration database is established to correlate temperature and acceleration changes in real-time. Finally, cavity length changes are compensated in real-time, ensuring the FPIA’s accuracy and stability in complex environments^32^.

### Mechanical characteristics of the sensor

The sensing diaphragm of the FPIA consists of a central proof mass and four groups of cantilever beams, which can be modeled as a typical elastic mechanical system. When external acceleration is applied along the Z-axis, the proof mass undergoes a small displacement due to inertia, resulting in a change in the length of the F–P cavity and consequently modulating the output interference signal. The axial response of this mass–spring system can be described by a second-order linear damping model, with the displacement expressed as^33^:

5 $$z(t)={X}_{0}{e}^{-\beta t}\,\sin (2\pi {f}_{n}t+\varphi )-{S}_{0}a$$

Here, *X*₀ and *φ* are constants representing the initial vibration state; *β* is the damping coefficient; *a* is the applied acceleration; *S*₀ denotes the static displacement sensitivity induced by acceleration; and *f*ₙ is the resonant frequency of the system, which is determined by the effective mass and the effective elastic stiffness of the system, and can be expressed as^34^:

6 $${f}_{n}=\frac{1}{2\pi }\sqrt{\frac{{k}_{eff}}{{m}_{eff}}}$$

Here, *m*_eff_ is the effective mass of the sensing diaphragm, and *k*_eff_ is its effective elastic stiffness, which can be expressed as:

7 $${k}_{eff}=\frac{\gamma E{W}_{c}{{T}_{c}}^{3}}{{{L}_{c}}^{3}}$$

Here, *W*_c_, *T*_c_, and *L*_c_ denote the width, thickness, and length of the beams, respectively; *E* is the Young’s modulus of silicon; and *γ* is the number of beams.

At the initial stage of excitation, the sensor structure exhibits a damped vibration process, which gradually decays to a steady-state displacement of *S₀a* as energy dissipates. When the system reaches steady state, the displacement of the proof mass under constant acceleration is proportional to the inertial force applied. Based on Newton’s second law and equilibrium conditions, *S₀* can be derived as follows:

8 $${S}_{0}=\frac{{m}_{eff}}{{k}_{eff}}$$

From Eqs. (6)–(8), the expressions for the sensitivity and resonant frequency of the FPIA are obtained as:

9 $${S}_{0}=\frac{({{L}_{m}}^{2}{T}_{m}\rho +\gamma {L}_{c}{W}_{c}{T}_{c}\rho ){{L}_{c}}^{3}g}{\gamma E{W}_{c}{{T}_{c}}^{3}},{f}_{n}=\frac{1}{2\pi }\sqrt{\frac{\gamma E{W}_{c}{{T}_{c}}^{3}}{({{L}_{m}}^{2}{T}_{m}\rho +\gamma {L}_{c}{W}_{c}{T}_{c}\rho ){{L}_{c}}^{3}}}$$

Here, *L*_m_ and *T*_m_ denote the side length and thickness of the proof mass, respectively; *ρ* is the density of silicon; and *g* is the gravitational acceleration. It can be observed that there is a clear trade-off between the sensitivity and resonant frequency of the FPIA: increasing sensitivity generally reduces the resonant frequency, and vice versa. Therefore, in the design of the FPIA, both sensitivity and dynamic response must be considered, and the structural parameters should be selected according to application requirements to achieve a balance between measurement accuracy and bandwidth.

In addition, the symmetric arrangement of multidirectional beams combined with the central proof mass ensures that applied transverse acceleration is evenly distributed in multiple directions. This design optimizes the stress distribution, reduces bending strain caused by transverse acceleration, and effectively lowers cross-axis sensitivity. Owing to the symmetric beam configuration, external forces are not concentrated in a single direction, thereby reducing local deformation and enhancing robustness against transverse disturbances.

Sensitivity and resonant frequency are two key performance indicators of the FPIA, both strongly influenced by the geometric parameters of the structure (such as beam length, width, and thickness) and material properties (such as density and Young’s modulus). To achieve optimal balance, the sensing diaphragm was precisely modeled using the SolidWorks platform, and finite element analysis and optimization of the key structural parameters were performed. This process yielded design parameters that meet the requirements of high sensitivity and wideband response.

**Fig. 2:**
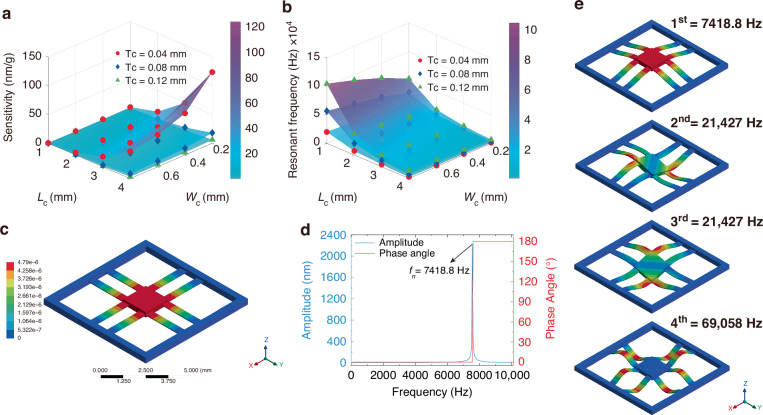
Sensitivity, resonant frequency, and mechanical characteristics of the FPIA. **a** Response of sensitivity to variations in beam parameters. **b** Response of resonant frequency to variations in beam parameters. **c** Displacement contour map. **d** Frequency and phase response. **e** Simulation results of the 1st to 4th order modes

As shown in Fig. 2a, 2b, beam thickness is the most critical factor. Reducing beam thickness significantly decreases structural stiffness, which greatly increases the displacement response of the FPIA under unit acceleration, resulting in a marked improvement in sensitivity. At the same time, the reduction in stiffness also lowers the resonant frequency of the system. Beam length has a secondary effect: increasing it enlarges the effective mass and compliance of the structure, which enhances sensitivity while reducing resonant frequency, though the trend is less pronounced than for beam thickness. In contrast, beam width has the weakest influence, only slightly affecting sensitivity under certain parameter combinations and showing negligible impact on frequency. Therefore, in FPIA structural design, beam thickness should be adjusted first to achieve initial optimization of performance indicators, beam length should be used for fine-tuning, and beam width should be adjusted as needed to satisfy structural or fabrication constraints. In this way, a reasonable trade-off between sensitivity and bandwidth can be realized.

Considering the combined effects of geometric parameters on sensing performance, along with the constraints imposed by PWR operating conditions on device size, installation space, and environmental adaptability, this study optimized the trade-off between sensitivity and resonant frequency. The final structural parameters of the sensing diaphragm are summarized in Table 1. Based on these parameters, the sensitivity and resonant frequency of the FPIA were calculated from Eq. (9) to be 4.889 nm/g and 7128.8 Hz, respectively.

**Table 1:** Parameters of the FPIA

Symbol	Value	Description
*L*_o_	8 mm	Side length of the outer frame of the sensing diaphragm
*L*_i_	7.2 mm	Side length of the inner frame of the sensing diaphragm
*L*_c_ *W*_c_ *T*_c_ *L*_m_ *T*_m_ *E* *ρ* *ν* *g*	2.7 mm 0.5 mm 0.06 mm 1.8 mm 0.35 mm 169 Gpa 2329 kg/m^3^ 0.27 9.81 m/s ^2^	Length of the cantilever beam Width of the cantilever beam Thickness of the cantilever beam Side length of the proof mass Thickness of the proof mass Young’s modulus of silicon Density of silicon Poisson’s ratio of silicon Acceleration of gravity

In the simulation, an acceleration load of 1 g was applied to the top surface of the proof mass, while all four sides of the outer frame were fixed as boundary constraints. The structural region was meshed appropriately to ensure both accuracy and computational efficiency. Figure 2c shows the displacement contour under acceleration excitation. The maximum displacement, approximately 4.790 nm, occurs at the center of the proof mass. From this result, the static sensitivity of the FPIA was calculated to be 4.790 nm/g, indicating that the structure responds effectively to acceleration signals.

Furthermore, harmonic response analysis was performed to obtain the amplitude–frequency and phase–frequency curves of the structure, as shown in Fig. 2d. When the excitation frequency approached 7418.8 Hz, the response amplitude reached its peak and the phase angle exhibited a transition, confirming that this frequency corresponds to the first-order resonant frequency of the structure. This result demonstrates that the FPIA achieves high sensitivity while maintaining strong dynamic response, providing a theoretical basis for subsequent bandwidth design and dynamic signal acquisition. Figure 2e shows the simulation results for the 1st to 4th resonance frequencies of the FPIA. The higher-order modes have significantly higher frequencies than the 1st mode, indicating no impact on the 1st mode’s response.

Finite element analysis (FEA) results show that the designed FPIA has a sensitivity of 4.790 nm/g and a resonant frequency of 7418.8 Hz. These values are in close agreement with the theoretical calculations (4.889 nm/g and 7128.8 Hz), validating the reliability of both the simulation model and theoretical analysis. The small discrepancies may be attributed to simplified treatment of boundary conditions and material parameters in the simulation, but the overall error remains within an acceptable range. It is worth noting that the FIV frequency of PWR heat transfer tubes typically remains below 50 Hz. Therefore, the designed FPIA can fully satisfy the measurement requirements and demonstrates strong applicability in practical engineering scenarios.

### Fabrication and packaging

**Fig. 3:**
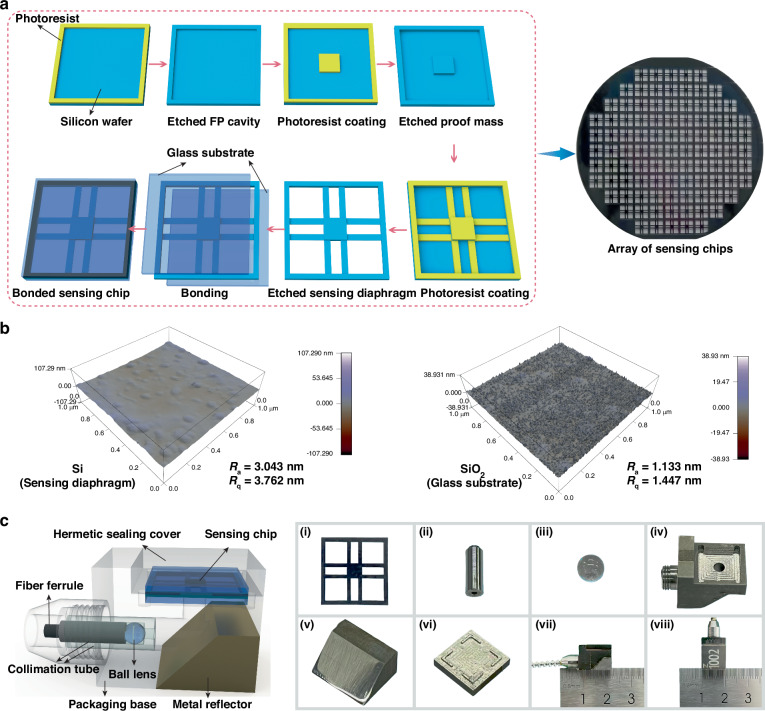
MEMS processes and Packaging of FPIA. **a** Wet etching process. **b** AFM images of the sensing diaphragm and the glass substrate. **c** Packaging process of FPIA. (i) Sensing chip; (ii) Collimation tube; (iii) Ball lens; (iv) Packaging base; (v) Metal reflector; (vi) Hermetic sealing cover; (vii) Side view of the FPIA; (viii) Top view of the FPIA

As shown in Fig. 3a, the sensing chip was fabricated using a MEMS-based process. First, the top and bottom surfaces of the silicon wafer were cleaned and dried to remove organic residues, particles, and other contaminants, thereby improving the adhesion between the photoresist and the substrate. A layer of photoresist was then spin-coated onto the wafer, followed by the first photolithography exposure using a mask to define the F–P cavity pattern. After wet etching, a precise optical cavity structure was formed on the wafer surface. Wet etching offers advantages such as simple equipment, fast etching rate, and minimal surface damage, making it particularly suitable for fabricating structures with dimensions larger than 1 μm^35,36^.

After cavity etching, two additional rounds of “photoresist removal, re-coating, and photolithography” were performed to define and wet-etch the proof mass and cantilever beam structures, ultimately forming the sensing diaphragm. The processed diaphragm was then bonded with two glass substrates to form a three-layer structure. High-temperature silicon–glass anodic bonding was employed for packaging, resulting in a sealed cavity with good mechanical strength, yielding a high-temperature accelerometer sensing chip.

The three-layer design of the sensing chip improves the FPIA’s consistency and repeatability, ensuring stability in high-temperature and high-pressure environments. We selected thermally stable materials and conducted rigorous testing to ensure reliability. The central diaphragm is protected by glass substrates, preventing overload damage and reducing temperature effects on performance.

Figure 3b shows the AFM (Atomic Force Microscope) test results for the surface roughness of the sensing diaphragm (proof mass) and the glass substrate after wet etching. The results indicate that both surfaces’ *R*_a_ (Arithmetic Average Roughness) and *R*_q_ (Root Mean Square Roughness) are within a small range. The *R*_a_ of the sensing diaphragm is 3.043 nm, and the *R*_q_ is 3.762 nm. The *R*_a_ of the glass substrate is 1.133 nm, and the *R*_q_ is 1.447 nm, indicating that the surfaces are smooth and can effectively ensure the optical performance of the FPIA.

The FPIA developed in this work was specifically designed to meet the measurement requirements in the confined, high-temperature, and high-pressure environment inside a PWR. As shown in Fig. 3c, to accommodate the special installation requirement where the FIV direction of the SG heat transfer tubes is perpendicular to the sensor axis, a metal reflector with a 45° inclination was integrated inside the FPIA structure. This configuration bends the optical path by 90°, effectively avoiding excessive bending of the optical fiber and enabling a compact design. To enhance reflectivity, the surface of the reflector was processed with a high-reflectivity metal polishing treatment. In addition, a high-transmittance ball lens was placed in front of the reflector to collimate the diverging light beam, thereby improving optical focusing and enhancing the overall optical performance of the FPIA.

The FPIA features high-temperature sealing using laser welding, ensuring optical component sealing under harsh conditions. The fiber ferrule is fixed by collimation sleeves and the ball lens is made of quartz, which performs well under high temperatures and pressures. During long-term testing, the quartz lens showed no significant degradation. The chip is sintered above the package base with glass solder, and the metal reflector is made from GH2747 high-temperature alloy, offering strength and temperature resistance. The reflector is polished for improved high-temperature and oxidation resistance. The package base and cover are made from 4J29 Kovar alloy, ensuring thermal stability. A multi-layer protection structure ensures long-term stable operation under harsh conditions.

## Results

### Experimental setup

**Fig. 4:**
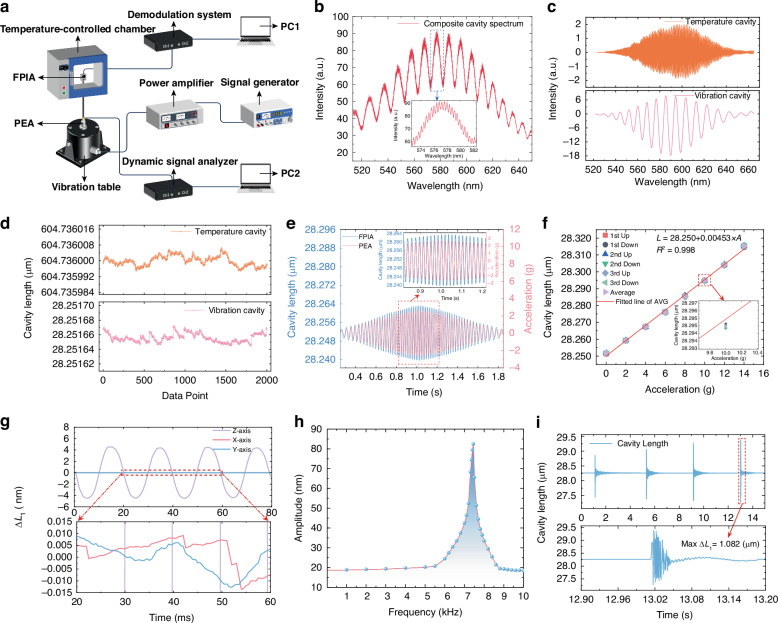
Experimental setup and room-temperature performance of the FPIA. **a** Schematic diagram of the experimental setup. **b** The interference spectrum of the composite cavity. **c** Spectrum of the temperature cavity and vibration cavity. **d** Cavity length variation of the FPIA during static test. **e** Dynamic performance of the FPIA. **f** The response of the FPIA at different accelerations. **g** Cross-axis response of the FPIA. **h** Frequency response of the FPIA. **i** Maximum measurement range of the cavity length by hammering experiments

Figure 4a shows the experimental system constructed for testing the FPIA. The setup mainly consists of a signal generator (SA-SG030), a power amplifier (SA-PA010), an electrodynamic shaker (SA-JZ005), a dynamic data analyzer (SA1804B), a custom-built fiber-optic demodulation system, a high-temperature chamber, a standard piezoelectric accelerometer (SAPC0100BD, PEA), and the FPIA under test. During the experiment, the signal generator produced excitation signals at specific frequencies, which were amplified by the power amplifier to drive the shaker and provide vibration excitation. The two sensors were rigidly connected to simultaneously receive the vibration signal, with the PEA serving as the reference device for calibrating the FPIA. The PEA output was acquired by the dynamic data analyzer, while the FPIA was placed in the high-temperature chamber, and its output signal was measured using the custom demodulation system. The demodulation system integrates a white-light LED source with a wavelength range of 500–700 nm, an optical coupler, a photodetector, and signal demodulation circuits. The measurement resolution reaches 0.02 nm, enabling high-precision detection of FPIA signals.

As shown in Fig. 4b, the demodulation system output provides the interference spectrum of the composite cavity after connection of the experimental devices. The interference fringes of the FPIA are clear and stable, exhibiting high contrast. As illustrated in Fig. 4c, equidistant wavenumber sampling and spectrum reconstruction allow complete decoupling of the temperature cavity and vibration cavity^37^. The static cavity lengths were determined to be approximately 604.736 μm for temperature cavity and 28.252 μm for vibration cavity, serving as critical baselines for subsequent temperature–vibration signal measurement and dynamic performance evaluation. Figure 4d shows the measured cavity length fluctuations of the FPIA under static conditions, where the maximum variations are 0.006 nm for the temperature cavity and 0.016 nm for the vibration cavity. The minimal fluctuations demonstrate the FPIA’s high static stability with a low noise floor.

### Room-temperature performance testing

To evaluate the dynamic performance of the developed FPIA, dynamic response tests were first conducted under room-temperature conditions and compared with the PEA. In the experiment, the signal generator was operated in sweep mode to apply acceleration signals to the FPIA, with the frequency linearly increased from 2 Hz to 50 Hz and then decreased back to 2 Hz. The response signals of both the FPIA and PEA were collected, and the frequency sweep results are shown in Fig. 4e. The results indicate that within the 2–50 Hz sweep range, the output waveform of the FPIA is highly consistent with that of the PEA, demonstrating that the developed FPIA possesses reliable dynamic performance and response characteristics.

To further determine the sensitivity of the FPIA, cavity length responses under different acceleration conditions were tested and analyzed. In the experiment, sinusoidal acceleration signals with a frequency of 50 Hz were applied, increasing from 0 g to 14 g in steps of 2 g and then decreasing back to 0 g with the same step size. The loading–unloading cycle was repeated three times, and the peak cavity length variation of the FPIA output under each load condition was recorded. The measurement results are shown in Fig. 4f. Linear fitting analysis indicates that the measured sensitivity of the FPIA is approximately 4.53 nm/g, with a coefficient of determination (*R*²) of 0.998, reflecting a highly linear response. Furthermore, the measured sensitivity is very close to the simulated result (4.790 nm/g), with minor discrepancies mainly attributed to dimensional tolerances and material parameter variations introduced during fabrication and assembly. Combining the measured sensitivity with the resolution of the demodulation system (0.02 nm), the acceleration measurement resolution of the FPIA was calculated to be 4.4 mg, demonstrating reliable measurement accuracy and stability.

To evaluate the cross-axis resistance of the FPIA, sinusoidal acceleration signals with a frequency of 50 Hz and an amplitude of 1 g were applied along the transverse (X- and Y-) axes, and the axial cavity length variations were measured. The results, shown in Fig. 4g, indicate that under X-axis and Y-axis excitation, the axial cavity length variations were only 0.301% and 0.281%, respectively. These results demonstrate that the effect of transverse acceleration on the axial output signal of the FPIA is negligible.

The strong cross-axis resistance is attributed to the symmetric arrangement of multidirectional beams in the sensing chip. The structure employs eight uniformly distributed cantilever beams to connect the central proof mass with the outer frame. These beams are symmetrically oriented in multiple directions, allowing transverse loads to be evenly transmitted and dispersed, thereby effectively reducing non-axial strain induced by transverse acceleration. The symmetric configuration of the cantilever beams plays a critical role in suppressing local torsion and controlling strain concentration, which significantly enhances the robustness of the device against cross-axis interference.

To test the resonant frequency of the FPIA, the acceleration amplitude was fixed at 4 g, and the frequency was gradually increased from 20 Hz to 10 kHz. The test results are shown in Fig. 4h. It can be observed that when the frequency exceeds 6000 Hz, the FPIA output rises sharply, peaking at approximately 7450 Hz, which is in close agreement with the simulated value of 7418.8 Hz.

Since the maximum acceleration provided by the shaker was limited to 20 g, which was insufficient for range testing of the FPIA, a hammer-impact method was adopted. Specifically, the FPIA was fixed onto a thin steel plate in a horizontal orientation, and strong impact signals were generated by striking the plate with a hammer. Multiple impacts were applied, and the output signals of the FPIA were recorded. A typical response is shown in Fig. 4i.

Under conditions where no saturation or distortion occurred in the output signal, the maximum cavity length variation of the F–P cavity reached ±1.082 μm. Based on the previously measured sensitivity, the maximum measurable acceleration range of the FPIA was calculated to be ±238 g, demonstrating its reliable capability for high-range shock response.

### High-temperature performance testing

**Fig. 5:**
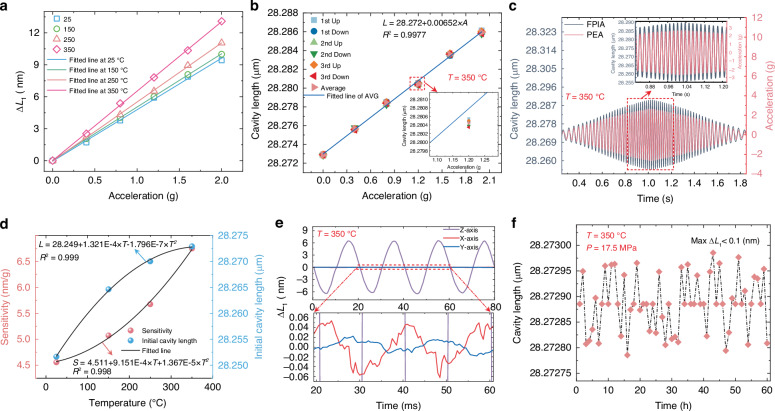
High-temperature and high-pressure performance of the FPIA. **a** Acceleration response across different temperatures. **b** The response of the FPIA at different accelerations under 350 °C. **c** Dynamic performance of the FPIA at 350 °C. **d** Sensitivity and the initial cavity length at different temperatures. **e** Cross-axis response of the FPIA at 350 °C. **f** Stability test results of the FPIA

To evaluate the performance variation of the FPIA under different temperature conditions, temperature sensitivity tests were carried out on its acceleration response. The FPIA was placed inside a high-temperature chamber, with the temperature sequentially increased from 25 °C to 150 °C, and then raised to 250 °C and 350 °C. At each set temperature, the system was held for 1 h to ensure thermal equilibrium. After stabilization, sinusoidal acceleration signals with a frequency of 50 Hz, amplitude ranging from 0 to 2 g, and a step of 0.4 g were applied at each temperature point. The measured responses are shown in Fig. 5a.

At 350 °C, the same loading–unloading test method was applied, and three repeated experiments were conducted to ensure the reliability and consistency of the data. The results, shown in Fig. 5b, indicate that the measured sensitivity of the FPIA was approximately 6.522 nm/g, with a coefficient of determination (*R*²) of 0.9977, confirming that the FPIA maintains a stable linear response even at high temperatures. The increase in FPIA sensitivity can be attributed to changes in material properties at high temperatures, mainly manifested as a decrease in Young’s modulus and the thermal expansion effect, which makes the sensing diaphragm more flexible and able to respond more sensitively to external acceleration, thereby enhancing the sensitivity.

The dynamic performance of the FPIA is critical at elevated temperatures. To evaluate this, the sensor was placed in a high-temperature chamber, heated to 350 °C, and held for 1 h. Dynamic response tests were then carried out following the same procedures as at room temperature. The frequency sweep curve of the FPIA at high temperature is shown in Fig. 5c. The results demonstrate that the FPIA maintains reliable dynamic performance under high-temperature conditions.

Figure 5d illustrates the significant increase in the FPIA’s sensitivity with temperature. As the temperature rises from 25 °C to 350 °C, sensitivity increases from 4.53 nm/g to 6.522 nm/g, highlighting the temperature’s effect on sensor performance. Additionally, the initial cavity length shown in Fig. 5d exhibits an upward trend with increasing temperature, indicating that temperature fluctuations can introduce drift in the cavity length and affect the FPIA’s output.

These results reveal the significant impact of temperature on the output characteristics of the FPIA, which can lead to accumulated measurement errors and compromise accuracy and reliability in practical applications. Therefore, in addition to precise temperature measurement, establishing a temperature calibration model is essential to compensate for the effects of thermal variations, thereby ensuring the accuracy and reliability of the FPIA across different environmental conditions.

The cross-axis performance of the FPIA at elevated temperatures was also investigated. After being held at 350 °C for 1 hour, sinusoidal acceleration signals with a frequency of 50 Hz and amplitude of 1 g were applied along the transverse (X- and Y-) axes, and the axial cavity length variations were measured. The results, shown in Fig. 5e, reveal cross-axis sensitivities of 0.959% and 0.628%, respectively, which are slightly higher than the values at room temperature (0.301% and 0.281%) but still remain at a low level.

At high temperatures, the thermal expansion mismatch between structural materials and the resulting stress changes affect the FPIA’s geometric symmetry, thereby increasing the coupling effect of lateral disturbances on cavity length variation, leading to an increase in cross-axis sensitivity. Nevertheless, due to the use of a well-symmetrized design and thermally stable materials, the cross-axis sensitivity of the FPIA remains low even at 350 °C. These results confirm that the FPIA maintains robust resistance to cross-axis interference under high-temperature conditions, highlighting its application potential in complex environments.

To further verify the stability of the FPIA in high-temperature and high-pressure environments, the sensor was placed in the chamber at 350 °C with an applied pressure of 17.5 MPa. The device was operated continuously for 60 h, with cavity length variations recorded every hour. As shown in Fig. 5f, the cavity length variation Δ*L*₁ remained below 0.1 nm throughout the entire test, indicating stable and reliable performance under high-temperature and high-pressure conditions.

### Discussion and conclusion

This work proposes a high-temperature and high-pressure temperature-calibrated FPIA based on a composite cavity, offering an innovative solution for microvibration monitoring of SG heat transfer tubes in PWR environments. By introducing a composite cavity structure and a temperature calibration model, temperature compensation was achieved, significantly improving measurement accuracy. The symmetric arrangement of multidirectional cantilever beams and a central proof mass further optimized cross-axis suppression, enhancing reliability in multidirectional vibration monitoring.

To highlight the novelty and impact of this work, we have included a comparison table (Table 2) that contrasts the performance of our FPI-based optical accelerometer with other similar accelerometers from recent studies, focusing on key parameters such as sensitivity, resonant frequency, cross-axis sensitivity, and temperature compensation.

**Table 2:** Comparison with other FPI-based optical accelerometers

Ref.	Operating Conditions	Sensitivity	Resonant Frequency	Cross-axis Sensitivity	Temperature Compensation
^25^	800 °C	370 mv/g	3750 Hz	11%	None
^26^	400 °C	2.48 nm/g	10.008 kHz	1.22%	None
^27^	1500 °C	20.91 nm/g	2700 Hz	—	None
^28^	25 °C	4.91 nm	2100 Hz	4.57%	None
^29^	600 °C	38.66 nm/g	2446 Hz	4.09%	None
^30^	25 °C	12.397 nm/g	6624 Hz	6.91%	None
^31^	25 °C	11.55 dB re rad/g	357.5 Hz 1023.8 Hz	1.33%	None
This work	350 °C 17.5 MPa	4.53 nm/g	7450 Hz	0.281%	Composite cavity + Temperature calibration

Experimental results show that the FPIA exhibits a sensitivity of 4.53 nm/g, a resonant frequency of 7450 Hz, and cross-axis sensitivities as low as 0.281% at room temperature, demonstrating high dynamic response and anti-interference capability. Under high-temperature and high-pressure conditions of 350 °C and 17.5 MPa, the cavity length drift remained below 0.1 nm during a 60 h stability test, confirming long-term reliability under extreme conditions. Although cross-axis sensitivities increased slightly at high temperature (0.959% and 0.628% at 350 °C), the variation was not significant compared to room temperature, indicating that the FPIA retains strong robustness against cross-axis interference at elevated temperatures.

Given the superior cross-axis performance, future research will focus on extending the FPIA into a triaxial configuration to enable comprehensive three-axis vibration measurements. Another important direction is to further enhance the operating temperature by employing high-temperature-resistant materials, thereby broadening its application range and adaptability in extreme thermal environments. With these advancements, the FPIA will provide more comprehensive and efficient technical support for vibration monitoring.
